# Betaine in Metabolic Dysfunction-Associated Steatotic Liver Disease: Mechanisms of Action and Therapeutic Potential

**DOI:** 10.3390/antiox15091209

**Published:** 2026-09-20

**Authors:** Tatjana Radosavljevic, Jasmina Djuretic, Milica Brankovic, Janko Samardzic, Ivana Curuvija, Danijela Vucevic

**Affiliations:** 1 Institute of Pathophysiology “Ljubodrag Buba Mihailovic”, Faculty of Medicine, University of Belgrade, 11000 Belgrade, Serbia; danijela.vucevic@med.bg.ac.rs; 2 Department of Pathobiology, Faculty of Pharmacy, University of Belgrade, 11000 Belgrade, Serbia; jasmina.djuretic@pharmacy.bg.ac.rs; 3 Institute of Pharmacology, Clinical Pharmacology and Toxicology, Faculty of Medicine, University of Belgrade, 11000 Belgrade, Serbia; milicabrankovic137@yahoo.com (M.B.); jankomedico@yahoo.es (J.S.); 4 Institute of Virology, Vaccines and Sera “Torlak”, 11000 Belgrade, Serbia; ivanajakovljev1@gmail.com

**Keywords:** MASLD, betaine, insulin resistance, lipid metabolism, oxidative stress, mitochondrial dysfunction, inflammation, gut–liver axis, fibrogenesis

## Abstract

Metabolic dysfunction-associated steatotic liver disease (MASLD) is the most common chronic liver disease worldwide and a leading cause of advanced liver fibrosis, cirrhosis, and hepatocellular carcinoma. The disease develops through the interaction of multiple interconnected pathophysiological mechanisms, including insulin resistance, dysregulated lipid metabolism, oxidative stress, mitochondrial and endoplasmic reticulum dysfunction, chronic inflammation, gut–liver axis disruption, and progressive fibrogenesis. Betaine, a naturally occurring methyl donor, has been investigated as a potential therapeutic agent because of its diverse metabolic and cytoprotective effects. This review summarizes current knowledge on the mechanisms by which betaine may influence MASLD development and progression. Preclinical studies indicate that betaine improves insulin sensitivity and hepatic lipid homeostasis, reduces oxidative and endoplasmic reticulum stress, preserves mitochondrial function, mitigates inflammatory signaling, and limits hepatic fibrosis. In addition, growing evidence suggests that betaine contributes to the maintenance of gut–liver axis homeostasis, further supporting its beneficial effects on liver function. Although these findings are consistent across preclinical models, clinical evidence remains limited, and the therapeutic efficacy of betaine in patients with MASLD has not yet been established. Further well-designed clinical trials are required to determine its clinical value, optimal therapeutic strategy, and potential role in the management of MASLD.

## 1. Introduction

Metabolic dysfunction-associated steatotic liver disease (MASLD) is the leading cause of chronic liver disease worldwide, with a prevalence approaching 40% in adults and 11% in adolescents [1,2,3,4,5,6]. It encompasses a spectrum ranging from simple steatosis to metabolic dysfunction-associated steatohepatitis (MASH), fibrosis, cirrhosis, and hepatocellular carcinoma (HCC) [7,8,9,10]. Its pathogenesis involves metabolic, oxidative, inflammatory, and fibrogenic mechanisms, including insulin resistance, impaired lipid metabolism, mitochondrial and endoplasmic reticulum (ER) dysfunction, gut–liver axis dysregulation, and chronic inflammation [9,11,12,13,14,15,16,17,18,19,20,21,22,23,24,25]. Lifestyle modification remains the cornerstone of MASLD management [26,27,28,29], although long-term adherence is often challenging and therapeutic options remain limited. Given the multifactorial and multicellular nature of MASLD, therapeutic strategies targeting multiple interconnected metabolic, inflammatory, and fibrogenic pathways may be more effective than those directed at a single molecular mechanism. Betaine affects several of these processes, including insulin signaling, lipid and one-carbon metabolism, oxidative stress, and inflammatory responses, which may be relevant to its protective effects in MASLD.

Betaine, also known as N-trimethylglycine, is a naturally occurring zwitterionic derivative of the amino acid glycine, with the chemical formula (CH_3_)_3_N^+^CH_2_COO^−^. It was first identified in the 19th century in the juice of sugar beet (*Beta vulgaris*) and is widely found in plants, animals, and microorganisms [30,31,32]. Betaine is most abundant in beets, wheat germ, spinach, wheat bran, and aquatic invertebrates (including bivalve mollusks such as mussels, oysters, clams, and scallops) [33,34,35,36,37,38]. In addition to exogenous intake through food, betaine is generated endogenously in mammals via the irreversible mitochondrial oxidation of choline through a two-step pathway in the liver and kidneys. First, choline is oxidized to betaine aldehyde by choline dehydrogenase, which is subsequently converted to betaine by betaine-aldehyde dehydrogenase [30,31,33,34]. Betaine is widely distributed across tissues, with the highest concentrations observed in the liver and kidneys. As a stable and non-toxic product, betaine plays a key role in maintaining cellular and metabolic homeostasis [30,32,33]. Although betaine is not classified as an essential nutrient because it can be synthesized endogenously, its endogenous production is generally insufficient to meet daily physiological requirements [31,33,38]. Daily betaine intake in the general population typically ranges from 100 to 300 mg [34,39,40,41]. After absorption in the duodenum, plasma concentrations generally reach 20–70 μmol/L. According to European Union (EU) food safety evaluations, betaine was classified as a “novel food” in 2017. By 2019, it had also been recognized as a functional ingredient in sports nutrition products, and the EU officially authorized its use on the market, with data protection granted until 2024 [42]. In the United States, betaine is classified by the Food and Drug Administration (FDA) as a dietary supplement and is available without a prescription. Researchers in sports medicine are increasingly investigating natural products and synthetic small molecules that may mimic the effects of exercise. Compared with conventional pharmacological agents, natural compounds are generally considered safer and potentially more suitable for long-term use, making them promising candidates as exercise mimetics and anti-aging agents [43,44,45,46]. This review integrates current evidence on the molecular mechanisms through which betaine modulates insulin resistance, lipid metabolism, oxidative stress, mitochondrial dysfunction, endoplasmic reticulum stress, inflammation, fibrogenesis, and gut–liver axis homeostasis in MASLD.

### Literature Search Strategy

A literature search was performed using PubMed, Scopus, and Web of Science to identify relevant studies on the biological and therapeutic effects of betaine in MASLD. The search included different combinations of keywords: “betaine” or “trimethylglycine” and “MASLD” and “MASH”, and “fatty liver disease”, “betaine” and “insulin resistance” and “lipid metabolism” and “oxidative stress” and “mitochondrial dysfunction” and “endoplasmic reticulum stress” and “inflammation” and “fibrosis”, and “gut–liver axis” and “mice”, and “rats”. Preclinical and clinical studies, as well as relevant review articles, published in English were considered. Particular attention was given to recent studies addressing the molecular mechanisms of betaine and its potential therapeutic relevance to MASLD.

## 2. Physiological Role of Betaine

In mammals, betaine serves three key physiological and metabolic functions. First, it is an organic osmolyte, which helps to maintain normal cell volume under osmotic stress. Second, it provides protection against protein denaturation, and therefore functions as a “chemical chaperone”. Third, as a methyl donor, it facilitates the remethylation of homocysteine to methionine within the methionine cycle [32,33,38]. The major physiological and biological functions of betaine and their underlying mechanisms are summarized in Table 1.

### 2.1. Betaine as an Osmoprotectant and Chemical Chaperone

One of the physiological roles of betaine (especially glycine betaine) is its function as an organic osmolyte. Due to its zwitterionic structure, betaine exhibits osmoprotective properties and accumulates in cells exposed to osmotic stress, where it stabilizes macromolecules, protects enzyme activity, and maintains membrane integrity and volume homeostasis without affecting cellular metabolism, stability, and viability [30,33,47,48,49,50]. Specifically, betaine functions as an osmolyte that facilitates cellular adaptation to adverse osmotic conditions. Betaine is known as a “compatible osmolyte” because it belongs to a group of small organic solutes that can accumulate to high intracellular concentrations without disrupting protein structure or enzymatic activity, thereby allowing cell adaptation to osmotic stress and environmental stressors, including dehydration, high salinity, and temperature extremes [33,47,51,52,53]. This property is particularly important in hepatocytes, where fluctuations in nutrient flux and redox state impose substantial stress on protein folding and intracellular hydration. Also, this role is particularly significant in the kidney medulla, where osmotic gradients are extreme. Cellular uptake and accumulation of betaine are mediated primarily by betaine/GABA transporter 1 (BGT1; solute carrier family 6 member 12, SLC6A12), whose expression is induced under hypertonic conditions via tonicity-responsive enhancer-binding protein (nuclear factor of activated T cells 5) (TonEBP/NFAT5), a key transcription factor regulating osmotic homeostasis and cellular adaptation to hypertonic stress [54,55]. Specifically, TonEBP has been identified as an immunometabolic regulator involved in obesity, insulin resistance, and lipid homeostasis, while also modulating pro-inflammatory signaling pathways [55,56]. In addition to its osmoprotective role, betaine functions as a chemical chaperone that stabilizes protein structure under stress conditions. It prevents protein misfolding, aggregation, and denaturation by promoting native conformations, particularly during osmotic, thermal, and oxidative stress, when proteostasis is disrupted. Through these mechanisms, betaine contributes to cellular integrity and stress tolerance, especially in liver disorders and metabolic syndrome-related diseases [57,58].

### 2.2. Betaine as a Methyl Group Donor

One of the primary physiological functions of betaine is to act as a methyl group donor. Namely, betaine donates a labile methyl group to homocysteine to form methionine in a reaction catalyzed by betaine–homocysteine methyltransferase (BHMT). Methionine is subsequently converted to S-adenosylmethionine (SAM) by methionine adenosyltransferase (MAT). This reaction supports the regeneration of SAM, a universal methyl donor involved in DNA and histone methylation, phospholipid metabolism and neurotransmitter synthesis. After donating its methyl group, SAM is converted to S-adenosylhomocysteine (SAH), which is hydrolyzed by SAH hydrolase to homocysteine and adenosine. Homocysteine can be remethylated to methionine via two pathways: (i) the folate-dependent methionine synthase (MTR) pathway, which uses 5-methyltetrahydrofolate and vitamin B12, and (ii) the betaine-dependent BHMT pathway, which uses betaine as the methyl donor and is especially active in the liver and kidney. This remethylation completes the methionine cycle. When remethylation is limited or when SAM levels are high, homocysteine is directed into the transsulfuration pathway: cystathionine β-synthase (CBS) condenses homocysteine with serine to form cystathionine, which is then cleaved by cystathionine γ-lyase to cysteine. Cysteine is subsequently used for glutathione and protein synthesis [33,44,59]. Therefore, betaine plays a key role in supporting the methionine cycle, contributing to cellular homeostasis, epigenetic regulation, antioxidant defense, and multiple biosynthetic processes. Maintenance of this methylation capacity is crucial for metabolic functions, cellular homeostasis, and regulation of gene expression [33,44,59]. Betaine contributes to epigenetic and post-transcriptional regulation by maintaining SAM availability and limiting homocysteine accumulation. In doing so, it supports DNA and histone methylation, influencing transcriptional programs related to mitochondrial biogenesis, oxidative phosphorylation, and inflammation. Recent studies indicate that the BHMT pathway modulates RNA methylation (m6A), linking nutrient-derived methyl supply to post-transcriptional control of metabolic genes [60]. The intracellular availability of SAM, along with the SAM/SAH ratio, serves as a key indicator of cellular methylation capacity, integrating nutritional status, metabolic flux, and redox homeostasis [33,61,62]. In that regard, changes in SAM homeostasis provide a direct metabolic signal that can influence epigenetic and epitranscriptomic processes, including N6-methyladenosine (m6A) RNA modification. In the liver, betaine acts as a metabolic–epigenetic regulator because it maintains SAM availability and modulates m6A regulatory pathways, influencing changes in hepatic gene expression, mitochondrial biogenesis, and lipid homeostasis [63,64]. Yang et al. showed that betaine increased the SAM/SAH ratio and enhanced m6A enrichment on NLRP3 mRNA [65]. The SAM–m6A axis links metabolic state to RNA fate, because intracellular SAM levels, accumulation of SAH, and the SAM/SAH ratio are critical determinants of m6A deposition and turnover. Plant-derived bioactive compounds such as betaine may modulate this axis and could therefore have potential applications in disease prevention and therapy [63]. Betaine is a biologically active metabolite with various physiological roles, including osmoregulation, cytoprotection, methylation, and metabolic regulation. Its involvement in key biological pathways highlights its importance in maintaining health and preventing disease [33]. Numerous studies, both preclinical and clinical, indicate the preventive and therapeutic effects of betaine in cardiovascular [33,66,67,68], hepatic [33,69,70,71,72,73], renal [33,74,75,76,77,78], and metabolic disorders [33,79,80]. Future research should focus on elucidating the molecular mechanisms underlying the effects of betaine and exploring its potential clinical applications. In this context, betaine has attracted considerable attention because of its ability to modulate multiple processes involved in disease progression. Experimental studies have shown that betaine improves insulin sensitivity, attenuates hepatic lipotoxicity and oxidative and endoplasmic reticulum stress, suppresses inflammatory responses, preserves mitochondrial function, and supports one-carbon metabolism through its methyl donor activity. These coordinated effects have been associated with reduced hepatic steatosis, slower fibrosis progression, and a lower risk of advanced liver injury in preclinical models [69,70,71,72,81,82,83]. Numerous preclinical studies have demonstrated beneficial and modulatory effects of betaine in experimental models of MASLD (Table 1). However, clinical evidence is still limited, and further randomized controlled trials are needed to establish its efficacy and optimal use. Although many studies have explored individual mechanisms of betaine action, the interconnection of signaling pathways underlying its hepatoprotective effects requires further research.

**Table 1:** Summary of preclinical studies investigating the effects of betaine relevant to MASLD.

Experimental Model	Model/Intervention	Betaine Dose/Concentration and Treatment Duration	Main Findings
**Animal models**			
C57BL/6 mice	TAA-induced liver fibrosis	2% (*w*/*v*) in drinking water; 6 weeks (weeks 2–8)	Reduced TGF-β1 and PDGF-BB signaling, modulated MMP-2/MMP-9/TIMP-1, and decreased collagen I and III deposition [69]
C57BL/6 mice	TAA-induced liver fibrosis	2% (*w*/*v*) in drinking water; 6 weeks (weeks 2–8)	Reduced hepatocellular injury, oxidative stress, and inflammation [70]
C57BL/6 mice	Methionine–choline-deficient diet-induced NAFLD	1.5% (*w*/*v*) in drinking water; 6 weeks	Improved liver histology and reduced hepatic steatosis and hepatocellular injury [71]
C57BL/6 mice	Methionine–choline-deficient diet-induced fatty liver	1.5% (*w*/*v*) in drinking water; 6 weeks	Reduced oxidative stress, inflammation, and apoptosis and modulated autophagy and Akt/mTOR signaling [72]
C57BL mice	High-fat diet-induced insulin resistance and fatty liver	1% (*w*/*v*) in drinking water; 14 weeks (preventive) or final 4 weeks after 14 weeks of HFD (therapeutic)	Improved hepatic insulin signaling and insulin sensitivity, with increased IRS-1/Akt/GSK3β activation and hepatic glycogen content and reduced steatosis [84]
Obese mice	Diet-induced obesity; betaine supplementation	2% (*w*/*v*) in drinking water; 8 to 25 weeks	Improved glucose metabolism and glucose utilization in liver and skeletal muscle and reduced systemic inflammation [85]
ApoE−/− mice	High-fat diet-induced NAFLD	2% (*w*/*v*) in drinking water; 8 weeks	Reduced hepatic lipid accumulation through FGF10/AMPK signaling, suppression of lipogenesis, and enhancement of fatty acid oxidation [86]
Rats	Fructose-induced NAFLD	62.5, 125, or 250 mg/kg; 4 weeks (weeks 5–8)	Reduced hepatic steatosis and ER stress through regulation of LXRα/PPARα signaling [87]
Mice	High-fat diet-induced hepatic steatosis	1% (*w*/*v*) in drinking water; 13 weeks	Improved hepatic lipid and iron homeostasis, including regulation of ZIP14, FTL/FTH, ferroportin, HAMP, and BMP2/BMP6–SMAD signaling [88]
Mice	High-fat diet-induced MASLD	0.3% (*w*/*w*) in diet; from the start of the intervention	Reduced hepatic iron accumulation and lipid peroxidation and attenuated ferroptosis through activation of the Nrf2/GPX4 pathway [89]
ICR mice	CDAHFD-induced NAFLD	0.2–1% (*w*/*v*) in drinking water; 1 week; 0.5% used in mechanistic autophagy experiments	Enhanced autophagy and reduced hepatic lipid accumulation and ER stress [90]
Rats	High-fat diet-induced NAFLD	1% betaine in the diet; 3 weeks	Improved sulfur-amino acid metabolism and antioxidant status and reduced oxidative stress [91]
**Cell-based** **studies**			
Primary human hepatocytes	Insulin-resistant hepatocytes; betaine treatment	0.63–20 mM; 24 h	Increased IRS-1 and Akt activation without changes in insulin receptor expression or activation, supporting a post-receptor insulin-sensitizing effect [84]
AML12 hepatocytes	Palmitic acid-induced lipotoxicity and ferroptosis; betaine treatment	200 μM; 24–48 h	Reduced iron accumulation and lipid peroxidation and increased Nrf2/GPX4/SLC7A11 signaling, resulting in attenuation of ferroptosis [89]
L8824 fish hepatocyte cell line	Lipid-overload model; betaine treatment	400 μM	Reduced lipid accumulation and enhanced VLDL secretion through regulation of the HNF4α/MTTP pathway [92]
Human adipocytes	Hypoxia-induced inflammatory response; betaine treatment	250 μM; 8–20 h	Reduced expression of hypoxia-induced inflammatory adipokines, including IL-6 and TNF-α [93]

## 3. The Effects of Betaine in MASLD/MASH

### 3.1. Betaine Modulates Insulin Resistance and Lipid Metabolism in MASLD/MASH

**Figure 1:**
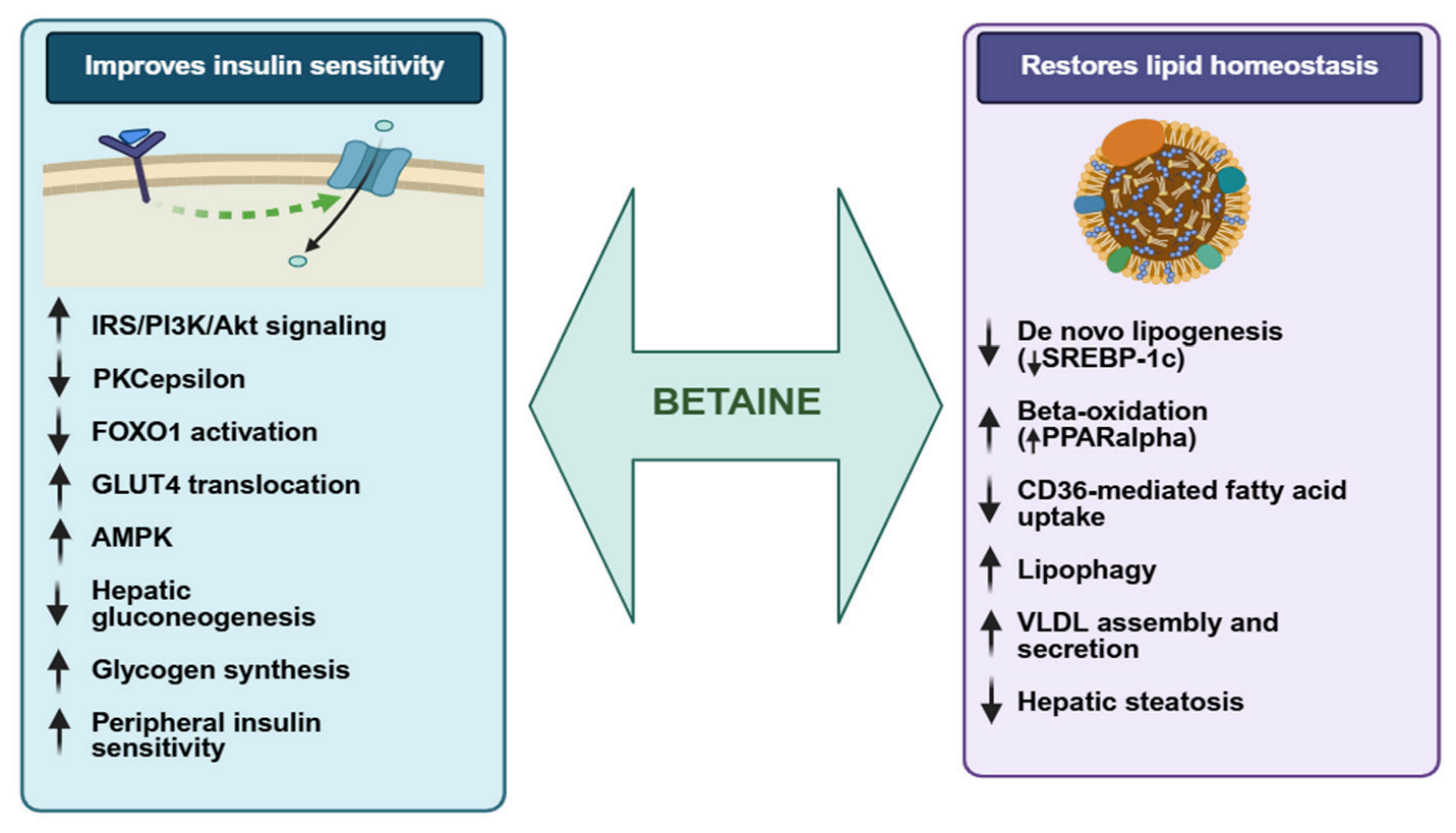
Effects of betaine on insulin sensitivity and lipid homeostasis in MASLD. The underlying molecular mechanisms are described in detail in the text. Abbreviations are defined in the Abbreviations list. Created in BioRender. Centar, I. (2026) https://BioRender.com/8em6ocg.

Insulin resistance and hepatic steatosis are major metabolic features of MASLD and represent important targets of betaine action. In adipose tissue, impaired insulin signaling increases lipolysis and the delivery of non-esterified fatty acids to the liver, thereby increasing the hepatic lipid burden. In the liver, insulin resistance is accompanied by persistent activation of lipogenic pathways, including SREBP-1c and ChREBP, which promote de novo lipogenesis and contribute to hepatic lipid accumulation [7,30,81,83]. Beyond triacylglycerol accumulation, excessive accumulation of lipid intermediates, particularly diacylglycerols and ceramides, can further impair insulin signaling. Diacylglycerol-mediated activation of PKCε interferes with the IRS-1/PI3K/Akt pathway, while ceramide accumulation can inhibit Akt signaling, further aggravating hepatic insulin resistance and metabolic dysfunction [17,94,95,96]. Oxidative stress and ER stress may additionally impair insulin signaling and contribute to metabolic dysfunction. Alterations in NADPH-dependent cytochrome P450 oxidoreductase (CPR) activity may additionally affect hepatic lipid metabolism and redox homeostasis [97]. Betaine improves insulin sensitivity through several complementary mechanisms involving hepatic insulin signaling, lipid metabolism, oxidative stress, mitochondrial function, one-carbon metabolism, and inflammatory signaling [84,85] (Figure 1).

#### 3.1.1. Effects of Betaine on Hepatic Insulin Signaling

Experimental evidence indicates that restoration of hepatic insulin signaling is one of the principal mechanisms underlying these effects. In both in vivo and in vitro models, betaine increased the activation of IRS-1, Akt, and glycogen synthase kinase 3β (GSK3β), accompanied by increased hepatic glycogen content [84]. Joselit et al. demonstrated that betaine reduced hepatic steatosis and enhanced insulin signaling in high-fat diet-fed mice [98]. In insulin-resistant primary human hepatocytes, betaine increased IRS-1 and Akt activation without altering insulin receptor expression or activation, indicating that its insulin-sensitizing effects occur predominantly at the post-receptor level. These findings indicate that restoration of IRS-1 signaling is a major mechanism by which betaine improves hepatic insulin sensitivity [84]. The precise mechanism through which betaine enhances IRS-1 tyrosine phosphorylation is still unclear. Possible explanations include reduced inhibitory serine phosphorylation of IRS proteins, attenuation of stress-activated kinases, improved membrane and protein stability, and altered methylation of genes regulating the signaling cascade. Clinical evidence in adults indicates that higher circulating betaine concentrations are inversely associated with fasting serum insulin, HOMA-IR, and other biomarkers related to diabetes [99].

The next mechanism by which betaine reduces insulin resistance is inhibition of hepatic gluconeogenesis and stimulation of glycogen synthesis. Namely, activated Akt phosphorylates and inhibits FOXO1, thereby suppressing the expression of gluconeogenic enzymes, including glucose-6-phosphatase, while promoting glucokinase activity and glycogen synthesis [84]. Betaine activates AMP-activated protein kinase (AMPK), a key metabolic sensor that shifts hepatic metabolism from lipid synthesis toward lipid oxidation. AMPK may enhance insulin signaling through crosstalk with the IRS-1/PI3K/Akt pathway, thereby contributing to FOXO1 inhibition. Also, betaine may activate AMPK, which enhances insulin signaling, glucose uptake, and fatty acid oxidation while reducing de novo lipogenesis. In hepatocytes, these effects are mediated in part by AMPK-dependent phosphorylation and inhibition of acetyl-CoA carboxylase, which lowers malonyl-CoA concentrations, promotes mitochondrial fatty-acid entry through carnitine palmitoyltransferase 1, and inhibits SREBP-1c-dependent hepatic lipogenesis [81]. Through the interconnected regulation of the IRS-1/PI3K/Akt/FOXO1 and AMPK signaling pathways, betaine may inhibit hepatic gluconeogenesis, increase glycogen synthesis, and improve lipid and cholesterol homeostasis, thereby reducing insulin resistance and hepatic steatosis [81,84]. Furthermore, AMPK may additionally reduce mTORC1–S6 kinase-mediated negative feedback on IRS proteins [100]. However, a direct causal link between betaine, AMPK activation, and reduced IRS inhibitory serine phosphorylation has not yet been clearly established.

#### 3.1.2. Effects of Betaine on Hepatic Lipid Metabolism

Another important mechanism by which betaine may improve hepatic insulin sensitivity is the attenuation of hepatic lipotoxicity, one of the principal drivers of impaired insulin signaling in MASLD. At the molecular level, betaine attenuates hepatic steatosis by disrupting the interaction between FOXO6 and peroxisome proliferator-activated receptor gamma (PPARγ), leading to inhibition of the PPARγ–CD36 axis and decreased transcription of lipogenic genes under hyperglycemic and diabetic conditions. Furthermore, betaine modulates hepatic inflammatory responses by inhibiting ROS-mediated FOXO6 signaling, a mechanism that may contribute to the reduced inflammation and improvement of insulin signaling in MASLD [101,102]. Betaine modulates hepatic lipid metabolism through several complementary mechanisms regulating lipid synthesis, utilization, and disposal. It suppresses de novo lipogenesis through downregulation of the major lipogenic transcription factors SREBP-1c and ChREBP, together with their downstream enzymes ACC, FASN, and SCD1. Betaine promotes mitochondrial fatty acid oxidation by activating AMPK and enhancing the PPARα/PGC-1α signaling axis, leading to increased CPT1 activity and improved mitochondrial oxidative capacity. Hepatic lipid overload is further alleviated by reduced CD36-mediated fatty acid uptake, attenuation of adipose tissue lipolysis, and enhanced very-low-density lipoprotein (VLDL) secretion through improved phosphatidylcholine synthesis [13,102]. In addition, betaine limits the excessive accumulation of lipid intermediates, including diacylglycerols and ceramides, stimulates lipophagy and autophagy, and preserves one-carbon metabolism via the BHMT/SAM pathway. Betaine also increases mitochondrial content and upregulates key regulators of mitochondrial biogenesis, including peroxisome proliferator-activated receptor gamma coactivator-1α (PGC-1α), nuclear respiratory factor-1 (NRF-1), and mitochondrial transcription factor A (TFAM) [81,94,102]. In ApoE−/− mice, betaine reduced hepatic lipid accumulation by inhibiting lipogenesis and enhancing lipophagy while promoting PPARα-dependent fatty acid oxidation [85,103].

Betaine further improves hepatic lipid metabolism by restoring gut–liver axis homeostasis. Preservation of intestinal barrier integrity limits endotoxin translocation into the portal circulation, reducing inflammatory signaling that drives hepatic lipid accumulation. In parallel, modulation of bile acid metabolism and FXR signaling may further improve hepatic lipid handling and reduce steatosis [104,105]. Through these coordinated mechanisms, betaine decreases hepatic triacylglycerol accumulation, improves mitochondrial function, and protects hepatocytes from lipotoxic injury, thereby attenuating the progression of MASLD [5,32,33,76,105]. Betaine inhibits the expression of lipogenesis-related genes through the FGF10/AMPK pathway, promotes fatty acid β-oxidation, and mitigates ER stress by regulating the expression of liver X receptor α (LXRα) and PPARα [72,86,87]. Enhanced mitochondrial function enables more efficient fatty acid β-oxidation, improves metabolic flexibility, and limits the excessive accumulation of lipid intermediates, particularly diacylglycerols and ceramides, which can contribute to impaired insulin signaling when present in excess. By decreasing the content of these lipotoxic intermediates, betaine may restore hepatic insulin sensitivity by reducing PKCε-mediated inhibition of IRS signaling and preventing ceramide-induced suppression of Akt activation [102]. Betaine may also modulate hepatic iron homeostasis and ferroptosis, providing an additional mechanism of hepatoprotection in MASLD. In HFD-fed mice, Li et al. showed that betaine improved hepatic iron homeostasis by regulating proteins involved in iron uptake, storage, and export, including ZIP14, FTL/FTH, and ferroportin, and by restoring HAMP expression and BMP2/BMP6–SMAD signaling. These effects were associated with changes in *HAMP* promoter methylation, suggesting an epigenetic contribution to the regulation of iron metabolism [88]. More recently, Zhai et al. demonstrated that betaine attenuated ferroptosis in HFD-fed mice and palmitic-acid-treated hepatocytes by reducing iron accumulation and lipid peroxidation and activating the Nrf2/GPX4 pathway. Increased GPX4 and SLC7A11 expression further supported the anti-ferroptotic effect of betaine [89]. Fat mass and obesity-associated (FTO) protein is a demethylase that plays a crucial role in demethylation. FTO-dependent demethylation inhibits the methylation of m6A, reducing lipid accumulation and energy metabolism [81,89,106]. Recent findings indicate that betaine may influence mitochondrial metabolism through an epitranscriptomic mechanism involving the BHMT–FTO–m6A–PGC-1α axis. Increased BHMT activity enhances one-carbon metabolism and improves cellular redox status, thereby supporting FTO-mediated m6A demethylation. Reduced m6A methylation of PGC-1α transcripts has been associated with increased PGC-1α expression and mitochondrial biogenesis, whereas epigenetic regulation of lipogenic genes, including SREBP1, FASN, and SCD1, contributes to reduced hepatic lipogenesis and triacylglycerol accumulation. These mechanisms improve mitochondrial biogenesis, fatty acid metabolism, and reduce hepatic lipotoxicity [63,75,76,106,107,108].

The hepatic epitranscriptome acts as a dynamic sensor of metabolic stress, integrating nutritional excess, systemic insulin resistance, and cellular stress into coordinated post-transcriptional responses promoting disease progression through positive feedback loops [109]. Additionally, betaine supplementation has been shown to increase hepatic and circulating fibroblast growth factor 21 (FGF21) levels, promoting white adipose tissue lipid oxidation and improving glucose homeostasis [33,75,110].

#### 3.1.3. Betaine, One-Carbon Metabolism, and Insulin Sensitivity

Another mechanism by which betaine may reduce hepatic insulin resistance involves the regulation of one-carbon metabolism and phosphatidylcholine synthesis. The liver’s high expression of betaine–homocysteine methyltransferase and its role in methionine metabolism position betaine as a crucial component of hepatic one-carbon metabolism [44]. Betaine is a methyl donor and serves as the substrate for BHMT, which catalyzes the remethylation of homocysteine to methionine. Methionine is subsequently converted to SAM, the key cellular methyl donor, thereby maintaining methylation capacity and epigenetic regulation [31,33,44,59]. In rats fed a high-fat diet, betaine supplementation upregulates the expression of key one-carbon metabolism enzymes, including BHMT, glycine N-methyltransferase (GNMT), and methionine adenosyltransferase (MAT), and restores hepatic methionine and SAM cellular levels [111]. Betaine lowers hepatic homocysteine and S-adenosylhomocysteine concentrations, supports the transsulfuration pathway and glutathione synthesis, and modulates the expression of genes involved in glucose and lipid metabolism [61]. It has also been shown that betaine supplementation in rats fed a high-fat diet upregulates the mRNA encoding BHMT, GNMT, and MGAT, all key enzymes of one-carbon metabolism involved in regulating fat metabolism [111]. In addition, the regulation of one-carbon metabolism is closely linked to hepatic lipid homeostasis. SAM provides methyl groups for phosphatidylethanolamine N-methyltransferase (PEMT)-mediated phosphatidylcholine synthesis, which is essential for the assembly and secretion of VLDL particles [31,44]. By preserving phosphatidylcholine availability, betaine facilitates hepatic triacylglycerol export, thereby limiting intracellular lipid accumulation and reducing hepatic lipotoxicity. Betaine is essential for DNA and RNA methylation, indicating an epigenetic mechanism whereby betaine regulates hepatic triacylglycerol metabolism [44,112]. Betaine serves as a methyl donor for DNA methylation in epigenetic regulation and provides a methyl group to participate in one-carbon metabolism to promote SAM formation [111]. Therefore, betaine, through epigenetic regulation, increases microsomal triglyceride transfer protein (MTTP) expression, further promoting VLDL secretion [31,44,98,109,113]. Also, improved phosphatidylcholine synthesis contributes to the maintenance of cellular and mitochondrial membrane integrity, thereby supporting mitochondrial function and reducing oxidative stress [31,92,114,115]. These coordinated effects of betaine may improve hepatic insulin sensitivity by preventing lipid-induced impairment of insulin signaling while simultaneously reducing homocysteine-mediated oxidative stress, endoplasmic reticulum stress, and inflammatory signaling, all of which contribute to IRS-1/PI3K/Akt dysfunction in MASLD [17,84,116,117]. Furthermore, recent evidence suggests that restoration of one-carbon metabolism by betaine may attenuate hepatic NLRP3 inflammasome activation, providing an additional mechanistic link between methyl-group metabolism, inflammation, and insulin resistance [44,118,119].

#### 3.1.4. Effects of Betaine on Oxidative Stress- and Inflammation-Associated Insulin Resistance

Another important mechanism by which betaine may improve hepatic insulin sensitivity is the mitigation of oxidative and ER stress, two closely interconnected processes that contribute to insulin resistance in MASLD. Excessive triacylglycerol accumulation in hepatocytes promotes mitochondrial dysfunction with ROS generation, which activates stress-sensitive signaling pathways, including c-Jun N-terminal kinase (JNK), inhibitor of κB kinase β (IKKβ), and TXNIP, thereby impairing IRS-1/PI3K/Akt signaling [120]. In addition, hepatic lipid overload disrupts protein folding within the ER, triggering the unfolded protein response (UPR). Persistent activation of the PERK–eIF2α, IRE1α–JNK, and ATF6 pathways promotes inflammation, hepatic gluconeogenesis, de novo lipogenesis, and inhibitory serine phosphorylation of IRS proteins, thereby exacerbating insulin resistance [121].

Betaine attenuates oxidative and ER stress through multiple interconnected mechanisms. By reducing hepatic lipotoxicity, betaine mitigates mitochondrial overload and ROS generation. In addition, by restoring one-carbon metabolism, betaine increases glutathione synthesis and cellular antioxidant defenses. Moreover, betaine enhances the activity of antioxidant enzymes, reduces lipid peroxidation, and protects mitochondrial membrane integrity. Furthermore, as an organic osmolyte and chemical chaperone, betaine stabilizes proteins and cellular membranes, thereby facilitating proper protein folding and limiting activation of the unfolded protein response in MASLD [32,122]. All these effects of betaine attenuate the activation of stress-responsive kinases and the TXNIP–NLRP3 inflammasome, thereby preserving insulin signaling through improved IRS-1 and Akt activation [118]. Studies in Western diet-induced models have demonstrated that betaine supplementation simultaneously attenuates oxidative stress, ER stress, inflammasome activation, and hepatic insulin resistance [105,123].

Given the close connection between oxidative stress and inflammatory signaling, the antioxidant properties of betaine contribute indirectly to its anti-inflammatory and insulin-sensitizing effects in MASLD. Inflammation-mediated impairment of insulin signaling is another mechanism of insulin resistance in MASLD. Chronic low-grade metabolic inflammation (metainflammation) is a major driver of both systemic and hepatic insulin resistance in MASLD. Hepatic lipid accumulation, lipotoxicity, de novo lipogenesis, oxidative stress, mitochondrial dysfunction, adipocyte-derived pro-inflammatory adipokines, and gut-derived endotoxins activate Kupffer cells and promote the recruitment of monocyte-derived macrophages, triggering inflammatory signaling within the liver and increasing the production of pro-inflammatory cytokines. Inflammatory cytokines activate several stress-related signaling pathways, including c-Jun N-terminal kinase (JNK), IκB kinase β (IKKβ)/nuclear factor-κB (NF-κB), and Janus kinase/signal transducer and activator of transcription (JAK/STAT) signaling [33,124]. Activation of JNK and IKKβ promotes inhibitory serine phosphorylation of insulin receptor substrate-1 (IRS-1), thereby disrupting downstream PI3K/Akt signaling and impairing insulin-mediated suppression of hepatic gluconeogenesis while reducing glycogen synthesis [124,125]. Also, inflammatory cytokines induce the expression of suppressor of cytokine signaling (SOCS) proteins, particularly SOCS3, which inhibit insulin receptor signaling by promoting IRS protein degradation and preventing its interaction with the insulin receptor. Inflammation also promotes activation of the TXNIP–NLRP3 inflammasome, leading to caspase-1 activation and the maturation of IL-1β and IL-18, which further amplify inflammatory signaling and exacerbate hepatic insulin resistance. Experimental studies in db/db mice and insulin-resistant hepatocytes indicate that inhibition of the FOXO1–TXNIP axis, accompanied by suppression of NLRP3 inflammasome activation, improves glucose tolerance, attenuates hyperinsulinemia, and restores hepatic insulin signaling [126]. Betaine may attenuate inflammation-mediated insulin resistance through multiple interconnected inflammatory pathways. Recent experimental evidence demonstrates that betaine reduces hepatic expression of pro-inflammatory cytokines, inhibits activation of the JNK, IKKβ/NF-κB, and TXNIP–NLRP3 signaling pathways, and promotes macrophage polarization toward an anti-inflammatory phenotype [119,127,128,129]. By suppressing chronic hepatic inflammation, betaine restores insulin signaling through the IRS-1/PI3K/Akt pathway and improves insulin sensitivity. The TLR4–MyD88–NF-κB pathway represents another mechanism linking inflammatory signaling to metabolic dysfunction, with its activation leading to the upregulation of TNF-α, IL-6, and MCP-1 [130]. Betaine may also suppress the HMGB1–TLR4 signaling pathway, another important inflammatory-mediated insulin resistance mechanism, leading to reduced circulating and hepatic levels of TNF-α, IL-6, and other pro-inflammatory mediators, thereby attenuating hepatic inflammation and improving insulin sensitivity [131,132,133].

### 3.2. Betaine Restores Redox, Mitochondrial, and Endoplasmic Reticulum Homeostasis Across the MASLD–MASH–HCC Continuum

Oxidative stress, mitochondrial dysfunction, and ER stress are closely interconnected processes that contribute to hepatocellular injury and MASLD progression [15,16,20,109,134,135,136,137]. Excessive hepatic lipid accumulation and metabolic dysfunction can increase mitochondrial ROS production and impair mitochondrial function, further aggravating oxidative stress and hepatocellular injury [138,139,140,141,142]. Beyond its insulin-sensitizing effects, betaine exerts multiple protective actions in MASLD/MASH/HCC by mitigating oxidative stress, preserving mitochondrial function, and alleviating ER stress.

#### 3.2.1. Betaine Mitigates Oxidative Stress

**Figure 2:**
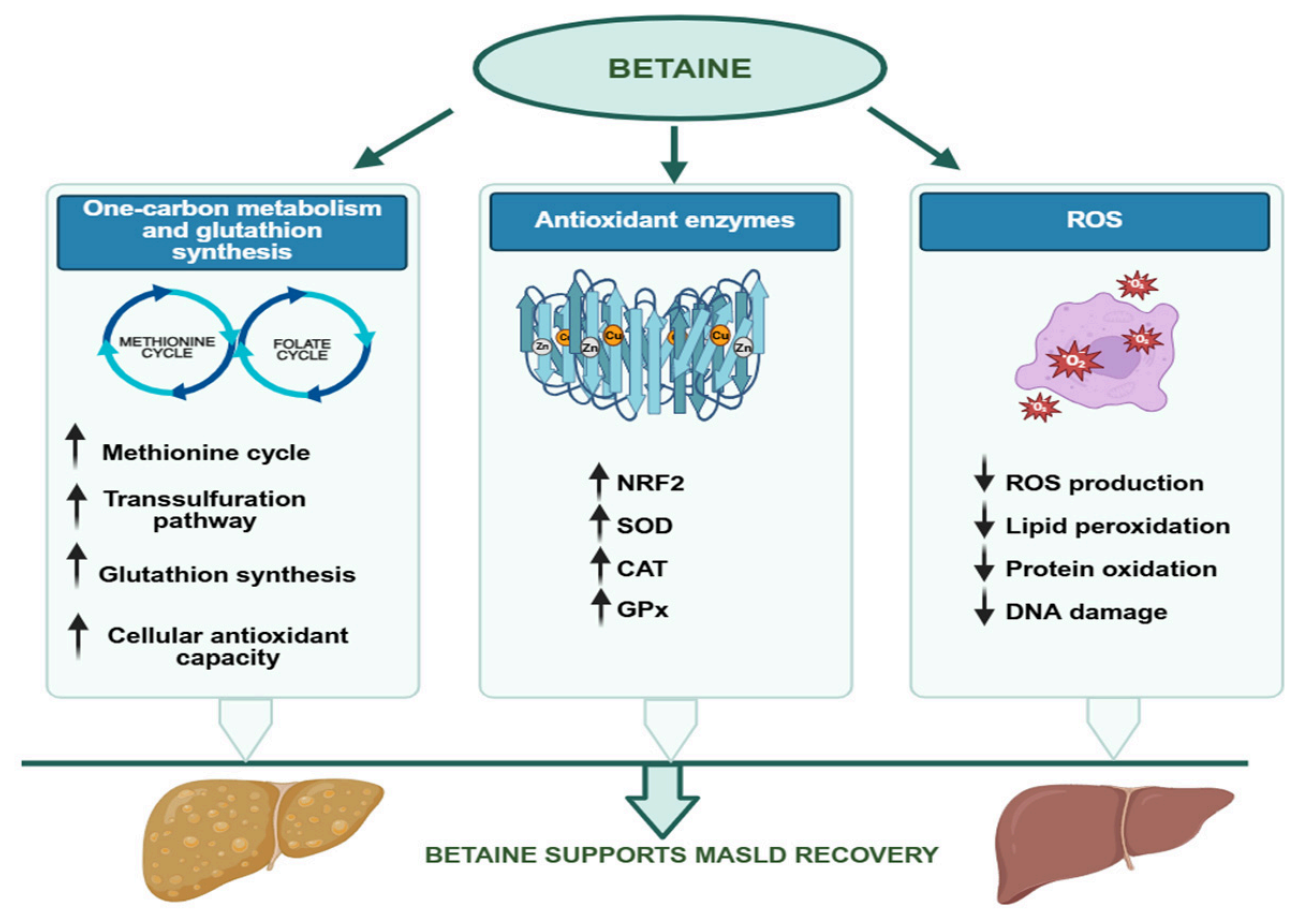
The effects of betaine on oxidative stress in MASLD. The underlying molecular mechanisms are described in detail in the text. Abbreviations are defined in the Abbreviations list. Created in BioRender. Centar, I. (2026) https://BioRender.com/jza3dmq.

Betaine exerts potent antioxidant effects in MASLD, ALD, and other chronic liver diseases [15,66,69,70,72,143]. As a methyl donor, it restores one-carbon metabolism and promotes glutathione synthesis through the methionine cycle and transsulfuration pathway, thereby enhancing cellular antioxidant capacity. In addition, betaine increases the activity of antioxidant enzymes, including superoxide dismutase (SOD), catalase (CAT), and glutathione peroxidase (GPx), which preserve membrane integrity, reduce lipid peroxidation, and protect hepatocytes from oxidative injury. Furthermore, betaine decreases hepatic ROS generation and reduces oxidative stress biomarkers, including malondialdehyde (MDA), 4-hydroxynonenal (4-HNE), protein carbonyls, which reflect oxidative modification of proteins, and advanced oxidation protein products (AOPPs), which are formed during oxidative modification and cross-linking of plasma proteins [15,31,44,66,69,70,72,74,122,143]. The effects of betaine on oxidative stress are summarized in Figure 2.

#### 3.2.2. Betaine Preserves Mitochondrial Homeostasis

Mitochondrial dysfunction is a hallmark of MASLD progression and is characterized by excessive ROS production, impaired fatty acid β-oxidation, ATP depletion, and defective mitochondrial quality control. In this regard, impaired mitochondrial dynamics, defective autophagy and mitophagy, enhanced apoptosis, disrupted proteostasis, and mtDNA instability lead to the accumulation of dysfunctional mitochondria with persistent ROS production and mitochondrial danger-associated molecular patterns (mtDAMPs) that can activate non-parenchymal liver cells and promote progression toward inflammation and fibrosis [15,20,144,145,146].

**Figure 3:**
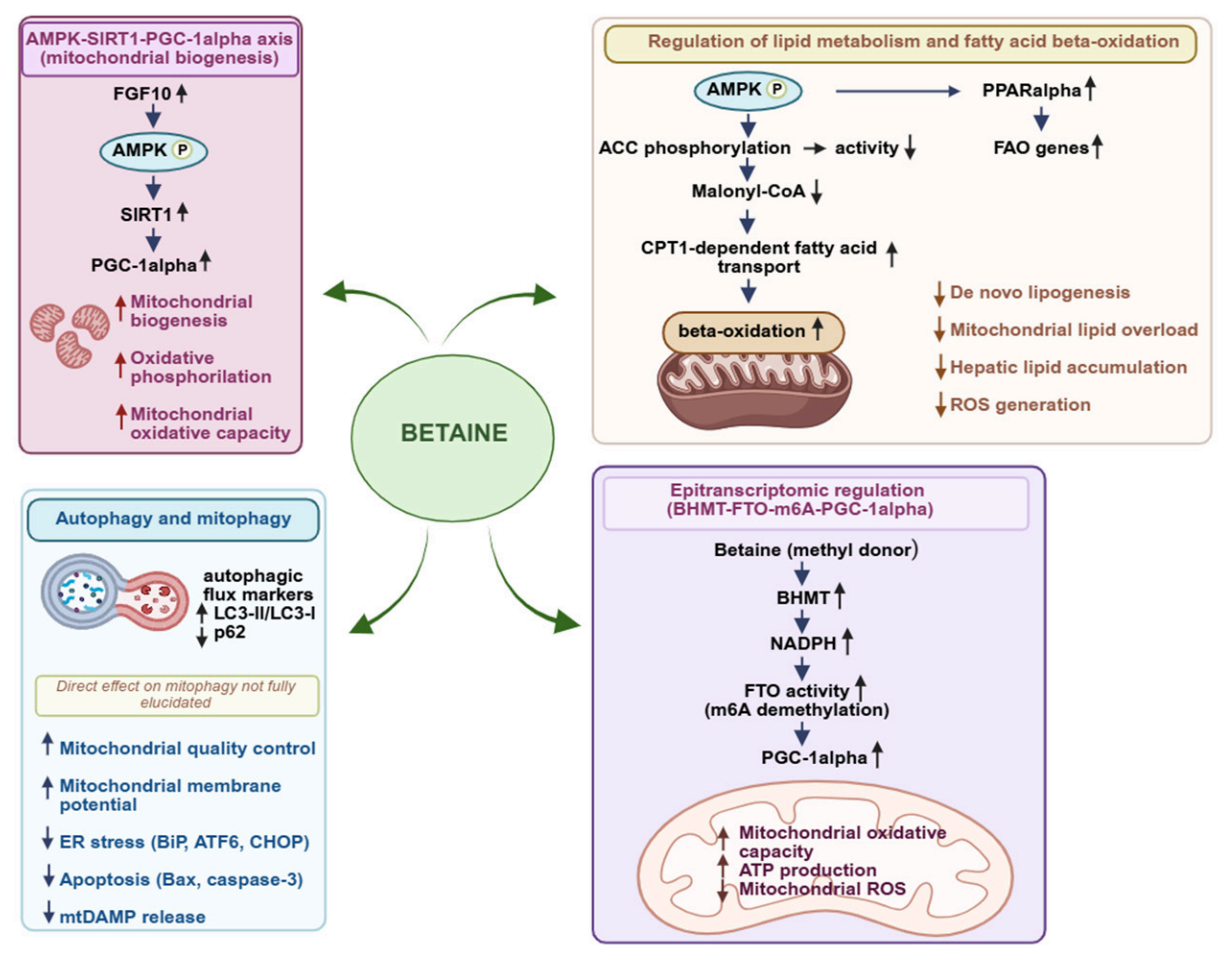
Betaine preserves mitochondrial function in MASLD. The underlying molecular mechanisms are described in detail in the text. Abbreviations are defined in the Abbreviations list. Created in BioRender. Centar, I. (2026) https://BioRender.com/ac3bq2l.

Beyond its antioxidant effects, betaine may preserve mitochondrial homeostasis through several interconnected mechanisms (Figure 3). AMPK plays a central role in maintaining mitochondrial function and cellular homeostasis by coordinating glucose and lipid metabolism together with autophagy/mitophagy. Through the integration of these interconnected pathways, AMPK maintains cellular energy balance and promotes adaptive responses to metabolic stress [90,147]. AMPK represents one of the principal molecular mediators of the mitochondrial protective effects of betaine. Experimental evidence indicates that betaine activates the AMPK–SIRT1–PGC–1α signaling axis, thereby promoting mitochondrial biogenesis, enhancing fatty acid β-oxidation, and restoring oxidative phosphorylation [32,81,90]. AMPK activation by betaine also inhibits acetyl-CoA carboxylase (ACC), lowers intracellular malonyl-CoA concentrations, facilitates CPT1-dependent mitochondrial fatty acid transport, and enhances PPARα-mediated transcription of β-oxidation-related genes, collectively reducing mitochondrial lipid overload and limiting ROS generation [91,148].

Chen et al. demonstrated that betaine supplementation induces fibroblast growth factor 10 (FGF10) secretion, leading to AMPK activation, suppression of hepatic lipogenesis, enhanced fatty acid oxidation (FAO), and ultimately reduced hepatic lipid accumulation, thereby identifying betaine as a potential therapeutic strategy in MASLD [86]. In addition, AMPK-dependent activation of autophagy and mitophagy facilitates the removal of dysfunctional mitochondria, preserves mitochondrial membrane potential, and maintains mitochondrial quality control [32,72,149,150]. Additionally, experimental studies have found that betaine activates autophagy, as evidenced by increased LC3-II/LC3-I and reduced p62 levels, thereby attenuating ER stress (BiP, ATF6, and CHOP), suppressing apoptosis (Bax and cleaved caspase-3), and reducing hepatic steatosis [71,72,149]. However, it remains unclear whether betaine directly enhances mitophagy or whether the observed improvement in mitochondrial quality control occurs secondary to reduced hepatic lipid accumulation and attenuation of oxidative stress.

Recent evidence further suggests that betaine regulates mitochondrial metabolism through epitranscriptomic mechanisms involving the BHMT–FTO–m6A–PGC-1α axis, thereby increasing mitochondrial oxidative capacity and ATP production while limiting mitochondrial ROS generation [76,107,108,109]. As a methyl donor, betaine also promotes glutathione synthesis and increases the activities of antioxidant enzymes. Experimental studies indicate that betaine supplementation reduces oxidative stress biomarkers, including MDA, 4-HNE, and AOPPs [32,70,72,151]. Overall, these effects of betaine reduce mitochondrial ROS generation, lipid peroxidation, protein oxidation, and mitochondrial DNA damage, thereby preserving mitochondrial integrity [32,81,91].

The gut–liver–mitochondria axis represents an additional link between systemic metabolic dysfunction and hepatic mitochondrial stress. Gut dysbiosis and microbiota-derived metabolites, including altered bile acids, short-chain fatty acids, lipopolysaccharide (LPS), and other pro-inflammatory mediators, modulate hepatic metabolism, ER stress, redox homeostasis, and mitochondrial function, contributing to the progression of MASLD. Betaine may restore gut–liver–mitochondria axis homeostasis by improving intestinal barrier integrity, reducing endotoxin translocation, and normalizing bile acid metabolism and short-chain fatty acid production. These changes reduce hepatic inflammatory signaling and metabolic stress, thereby indirectly preserving mitochondrial function and limiting oxidative stress [143,152,153,154].

In addition, betaine may indirectly preserve ER–mitochondrial communication by improving mitochondrial function, ATP production, and redox homeostasis, thereby supporting mitochondria-associated ER membrane (MAM) function and reducing mitochondrial Ca^2+^ overload and ER stress. Furthermore, by attenuating oxidative stress, preserving mitochondrial homeostasis, and enhancing mitochondrial quality control, betaine may attenuate mitochondria-mediated apoptosis, ultimately reducing hepatocyte injury, inflammation, and fibrogenesis [31,32,44,122,145,149]. Given the close interplay between mitochondria and the ER through MAMs, mitochondrial dysfunction contributes to ER stress and activation of the UPR. By improving mitochondrial function and redox homeostasis, betaine may also attenuate ER stress.

#### 3.2.3. Betaine Alleviates Endoplasmic Reticulum Stress

The ER serves as a central regulator of protein folding, phospholipid synthesis, calcium homeostasis, and intracellular stress signaling, making it particularly susceptible to disturbances in lipid metabolism and redox balance. Closely linked to oxidative stress and mitochondrial dysfunction, ER stress is a hallmark of MASLD progression and contributes substantially to hepatocellular injury. Disturbances in cellular redox homeostasis together with hepatic lipid overload impair protein folding, leading to the accumulation of unfolded and misfolded proteins within the ER and activation of the UPR [155,156]. These metabolic disturbances compromise ER homeostasis and activate the UPR through the PERK, IRE1α, and ATF6 signaling pathways. Although transient UPR activation is initially adaptive, persistent ER stress induces hepatocyte apoptosis, inflammation, and fibrogenesis, thereby facilitating MASLD progression. Therefore, chronic ER stress is now recognized as a key mechanism driving MASLD progression and MASH-associated HCC [155,156,157,158,159,160].

Among the proposed mechanisms, the role of betaine as a methyl donor is supported by the strongest experimental evidence. Through BHMT-mediated remethylation of homocysteine, betaine restores one-carbon metabolism, increases SAM availability, lowers homocysteine and SAH levels, and improves the SAM/SAH ratio. These metabolic effects preserve ER redox homeostasis, facilitate protein folding, maintain phosphatidylcholine synthesis and ER membrane integrity, and attenuate UPR activation [37,151].

Betaine may attenuate ER stress by reducing hepatic lipid overload and lipotoxicity [81], increasing antioxidant defenses [30], and preserving mitochondrial function [90], thereby restoring ER redox homeostasis, limiting ROS production and sustained PERK, IRE1α, and ATF6 activation, and interrupting the vicious cycle linking oxidative stress, mitochondrial dysfunction, and ER stress. Betaine appears to attenuate sustained PERK–eIF2α–ATF4–CHOP signaling, thereby limiting ER-stress-associated hepatocyte apoptosis [157].

Another mechanism underlying the protective effects of betaine against ER stress is the improvement of mitochondrial function and ER–mitochondrial crosstalk. The ER and mitochondria communicate through MAMs, which coordinate calcium homeostasis, lipid metabolism, and mitochondrial function [161,162,163]. Betaine may preserve ER–mitochondrial homeostasis by enhancing fatty acid oxidation, mitochondrial biogenesis, and ATP production while reducing ROS generation through activation of the AMPK–PGC-1α pathway. Although direct evidence for the regulation of MAMs by betaine is lacking, improved mitochondrial function is likely to indirectly stabilize ER–mitochondrial communication [32,81].

**Figure 4:**
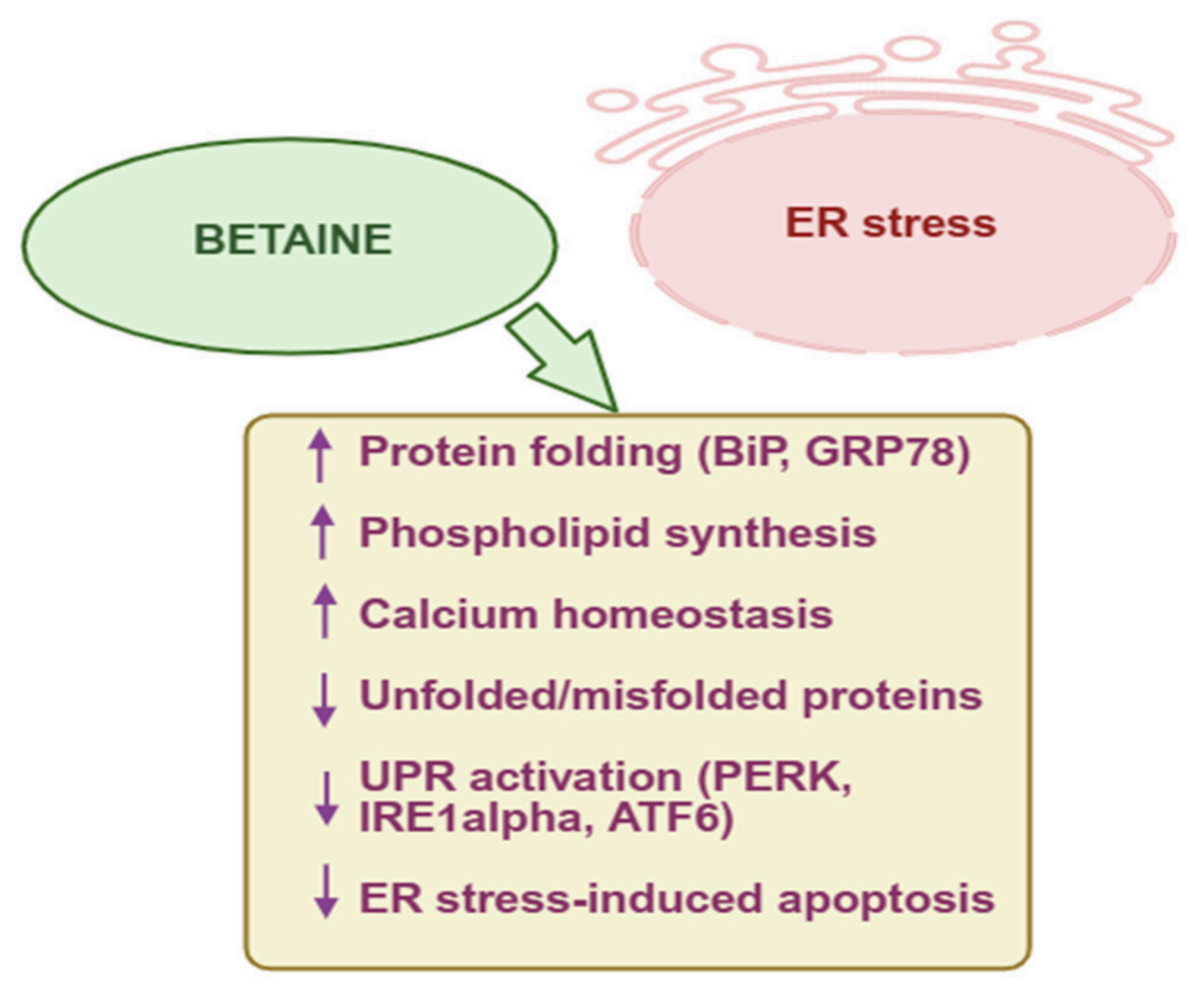
Effects of betaine on endoplasmic reticulum stress during MASLD progression. The underlying molecular mechanisms are described in detail in the text. Abbreviations are defined in the Abbreviations list. Created in BioRender. Centar, I. (2026) https://BioRender.com/wea4zcf.

Moreover, loss of ATF6 prevents steatosis caused by chronic ER stress but can also potentiate steatosis caused by acute ER stress. This demonstrates that ATF6 can play both protective and pathological roles in fatty liver disease [164]. Betaine indirectly regulates ATF6 activation by reducing the accumulation of misfolded proteins and restoring cellular redox and lipid homeostasis, as well as preserving adaptive chaperone responses, including BiP/GRP78-mediated protein folding. Furthermore, betaine may normalize IRE1α signaling by preventing persistent IRE1α–JNK activation while preserving the adaptive functions of the IRE1α–XBP1 pathway [155]. Therefore, attenuation of ER stress represents an important component of the hepatoprotective effects of betaine in MASLD. Despite substantial experimental evidence, the molecular targets through which betaine modulates the UPR remain largely undefined. Further investigations are needed to clarify these pathways and their contribution to the therapeutic potential of betaine. The effects of betaine on ER stress are summarized in Figure 4.

Overall, the hepatoprotective effects of betaine arise from the coordinated modulation of interconnected pathogenic mechanisms, including oxidative stress, mitochondrial dysfunction, ER stress, inflammation, and lipid dysregulation. Although substantial experimental evidence supports these effects, the precise molecular targets through which betaine regulates these pathways remain incompletely understood and warrant further investigation.

### 3.3. Betaine Mitigates Inflammation and Fibrosis During MASLD Progression

Chronic hepatic inflammation links metabolic injury to fibrogenesis during MASLD progression. Hepatic lipid accumulation, oxidative stress, mitochondrial dysfunction, and ER stress promote hepatocyte injury and the release of DAMPs, ROS, and pro-inflammatory mediators, leading to Kupffer cell activation and recruitment of monocyte-derived macrophages and other immune cells [165,166,167]. Extrahepatic factors also contribute to hepatic inflammation, particularly gut microbial dysbiosis and impaired intestinal barrier integrity, which increase the translocation of microbial products such as LPS into the portal circulation [166,168,169,170,171]. Persistent inflammatory signaling and the release of pro-inflammatory cytokines promote hepatic stellate cell activation and extracellular matrix deposition, driving the progression of MASH and fibrosis [168,172,173].

**Figure 5:**
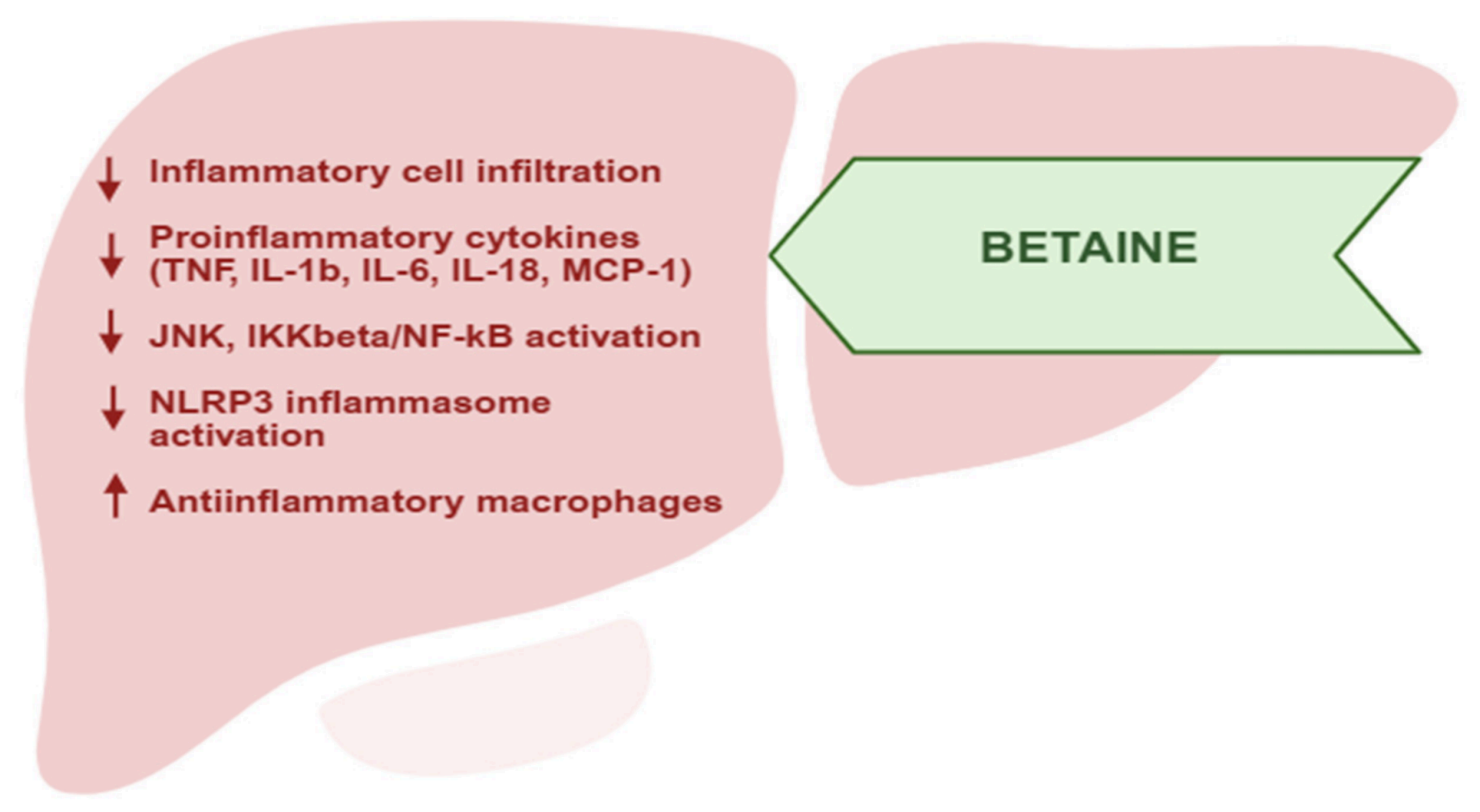
Effects of betaine on inflammation during MASLD progression. The underlying molecular mechanisms are described in detail in the text. Abbreviations are defined in the Abbreviations list. Created in BioRender. Centar, I. (2026) https://BioRender.com/9icul3n.

Betaine exerts anti-inflammatory effects primarily through attenuation of hepatic lipid accumulation and lipotoxicity, oxidative stress, mitochondrial dysfunction, and ER stress, thereby limiting activation of innate immune and inflammatory signaling pathways [33,44,72,105] (Figure 5). Accordingly, numerous experimental studies have demonstrated improved antioxidant status, reduced inflammatory cell infiltration, and partial restoration of metabolic homeostasis following betaine administration [72,105]. In a diet-induced obese mouse model, betaine treatment significantly enhanced glucose utilization efficiency in skeletal muscle and liver, and alleviated systemic inflammation [85]. Furthermore, betaine suppresses hypoxia-induced IL-6 and TNF-α mRNA expression in cultured human adipocytes. These observations indicate that, besides its beneficial effects, betaine may directly reduce adipose tissue inflammation, an important extrahepatic driver of chronic inflammation in MASLD [93]. Comparable anti-inflammatory effects of betaine were also observed in a high-fat diet-induced fish model, where activation of the SIRT1/SREBP-1/PPARα axis was observed, whereas proinflammatory signals, such as NF-κB, TNF-α, and interleukin-1 beta (IL-1β), were downregulated [174]. In addition to its indirect anti-inflammatory effects, betaine appears to influence several major inflammatory signaling pathways. Betaine-mediated suppression of the HMGB1/TLR4/NF-κB axis, together with inhibition of NLRP3 inflammasome activation, has been associated with reduced hepatic expression of TNF-α, IL-1β, IL-18, MCP-1, and other inflammatory mediators [33,81,131,132,133]. Because these signaling pathways are closely interconnected and activated simultaneously, the available evidence suggests that betaine exerts its biological effects through coordinated modulation of multiple pathophysiological mechanisms rather than selective inhibition of a single signaling cascade [17,105,131,132,133]. TLR4–MyD88–NF-κB signaling represents another inflammatory pathway relevant to metabolic dysfunction, with its activation promoting the expression of TNF-α, IL-6, and MCP-1 [130]. Furthermore, recent experimental studies demonstrated that betaine reduces hepatic expression of pro-inflammatory cytokines, suppresses JNK, IKKβ/NF-κB, and FOXO1–TXNIP–NLRP3 signaling, and promotes macrophage polarization toward an anti-inflammatory phenotype [118,119,127,128,129].

The gut–liver axis provides an additional site through which various therapeutic modalities may suppress hepatic and systemic inflammation. Recent evidence indicates that betaine preserves gut–liver axis integrity through multiple mechanisms [16,105,172]. One of the principal protective effects is the maintenance of intestinal barrier function by stabilizing tight junction proteins, thereby limiting translocation of bacterial products, including LPS, into the portal circulation and reducing hepatic inflammatory activation [51,175,176]. Betaine also modulates gut microbial composition, favoring the production of beneficial microbial metabolites, particularly short-chain fatty acids (SCFAs), which contribute to intestinal barrier integrity, mucosal immune homeostasis, and suppression of intestinal inflammation [17,177]. Given the central role of bile acids in regulating gut microbial ecology through FXR- and TGR5-dependent signaling, betaine-mediated modulation of bile acid metabolism may further preserve intestinal barrier function and gut–liver homeostasis [19,44,178,179]. Experimental studies additionally suggest that betaine modifies the intestinal osmotic environment, improves intestinal morphology and digestive function, and increases microbial diversity while reducing the production of pro-inflammatory microbial metabolites [180]. Taken together, these effects of betaine decrease intestinal permeability, limit endotoxin translocation, and attenuate both hepatic and systemic inflammation [51,181]. Furthermore, betaine exerts additional anti-inflammatory effects within the intestine by upregulating IL-4, downregulating TNF-α expression in the small intestinal mucosa, and increasing the production of secretory immunoglobulin A (sIgA), an important component of mucosal immunity involved in the neutralization of pathogens and other luminal antigens [105,182,183,184]. Betaine also modulates the gut microbiota by increasing the abundance of beneficial bacteria while reducing harmful bacteria [185]. In addition, inhibition of inducible nitric oxide synthase (iNOS) expression decreases nitric oxide (NO) production, thereby attenuating intestinal inflammation and further preserving gut barrier function [17,105].

**Figure 6:**
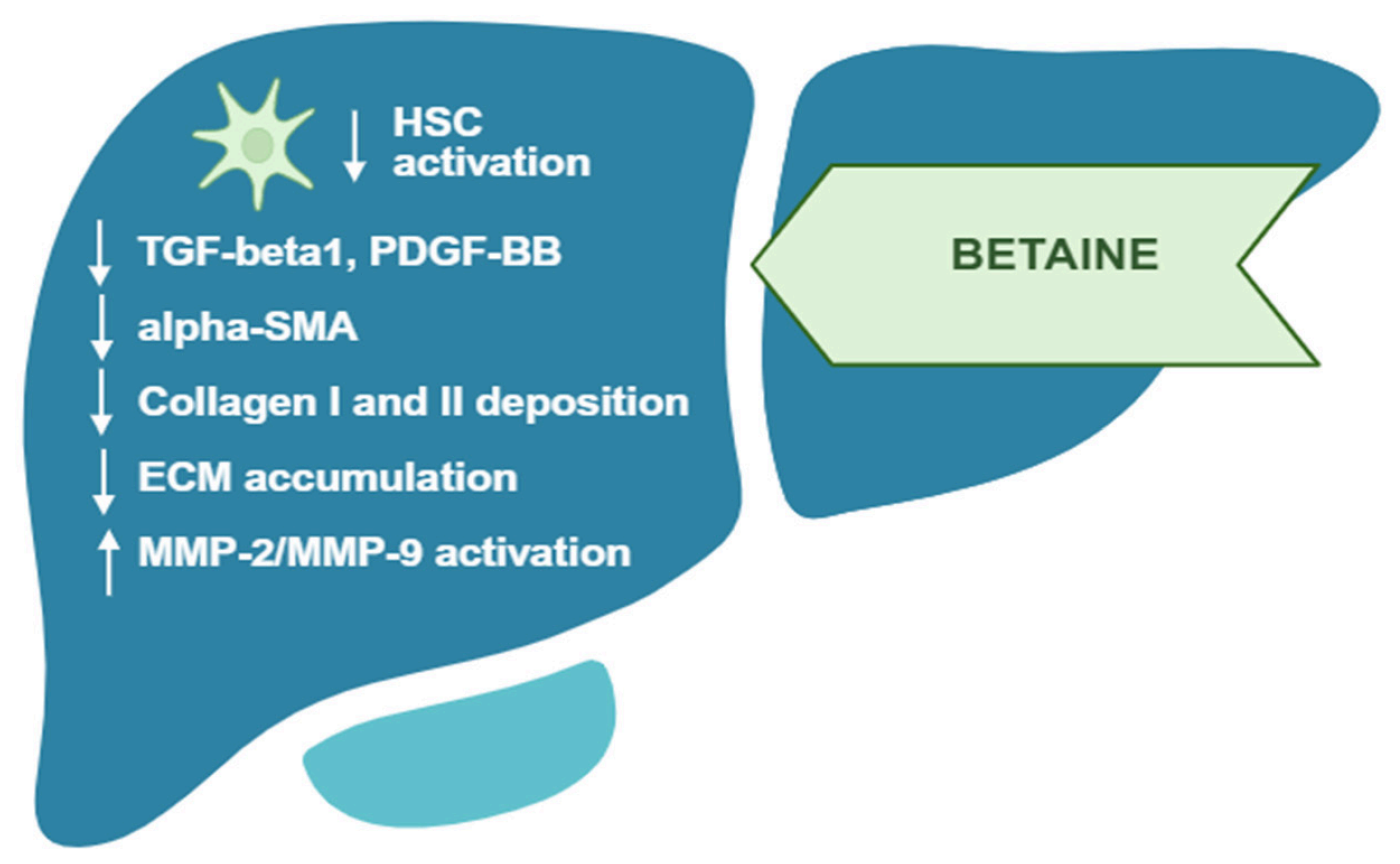
Effects of betaine on hepatic fibrogenic pathways during MASLD progression. The underlying molecular mechanisms are described in detail in the text. Abbreviations are defined in the Abbreviations list. Created in BioRender. Centar, I. (2026) https://BioRender.com/mjxkzj0.

Betaine mitigates MASLD progression through its antifibrotic effects (Figure 6). Current evidence suggests that these effects primarily reflect attenuation of the metabolic and inflammatory drivers of fibrogenesis rather than selective inhibition of a single profibrotic pathway [19,44,105]. By reducing hepatic lipid accumulation, oxidative stress, endoplasmic reticulum stress, mitochondrial dysfunction, and chronic inflammation, betaine limits the stimuli responsible for HSC activation and excessive ECM deposition. In parallel, betaine improves methionine metabolism and methyl-group availability through the BHMT-dependent remethylation pathway, thereby preserving SAM homeostasis and reducing epigenetic alterations associated with fibrogenesis. Experimental studies have consistently demonstrated reduced hepatic expression of transforming growth factor-β1 (TGF-β1), platelet-derived growth factor-BB (PDGF-BB), α-smooth muscle actin (α-SMA), collagen types I and III, and other extracellular matrix components following betaine administration [186,187]. Similar findings were obtained in our thioacetamide-induced mouse model, in which betaine attenuated TGF-β1 and PDGF-BB expression, modulated the MMP-2/MMP-9/TIMP-1 axis, and reduced collagen types I and III deposition [69]. Recent evidence further suggests that betaine may interfere with several processes involved in fibrogenesis, including macrophage-mediated inflammatory signaling and ECM remodeling [187,188], although its effects on epithelial–mesenchymal transition and other profibrotic mechanisms remain to be established.

An important consideration in interpreting the preclinical evidence is the relationship between physiological betaine concentrations and the doses used experimentally. Circulating betaine concentrations are generally in the low micromolar range (approximately 20–70 μmol/L) [34,39,40,41], whereas intracellular concentrations vary considerably among tissues and may be substantially higher because betaine can accumulate intracellularly as a compatible organic osmolyte [33,47,51,52,53]. In vitro studies relevant to MASLD have used betaine concentrations ranging from approximately 0.2 to 20 mM, while animal studies have employed markedly different supplementation regimens, including approximately 0.2–2% betaine in drinking water or diet and, less commonly, defined doses expressed as mg/kg (Table 1). Notably, mechanistic effects have been observed at relatively low concentrations, including modulation of inflammatory responses and ferroptosis at 200–250 μM, whereas restoration of insulin signaling in insulin-resistant human hepatocytes was examined over a substantially broader concentration range (0.63–20 mM) (Table 1). Differences in experimental models, doses, and treatment duration make direct comparisons between studies difficult. Future dose–response studies should therefore examine whether circulating and hepatic betaine concentrations are associated with specific molecular and metabolic effects.

Despite substantial preclinical evidence supporting the hepatoprotective effects of betaine, clinical evidence in MASLD remains limited. In a randomized placebo-controlled trial in patients with biopsy-proven NASH, betaine supplementation (20 g/day for 12 months) did not significantly improve serum aminotransferases, histological disease activity, fibrosis, or insulin concentrations compared with placebo [189]. More recent pilot trials in patients with non-cirrhotic MASLD and elevated ALT showed that lower doses of betaine (2–8 g/day for 12–24 weeks) reduced ALT and AST concentrations and several circulating markers of liver injury and fibrosis, whereas 1 g/day was ineffective [190]. However, the small sample sizes and limited clinical endpoints preclude definitive conclusions regarding therapeutic efficacy. An additional consideration for clinical translation is betaine insufficiency. Unlike classical micronutrient deficiencies, there is currently no established biochemical threshold defining betaine deficiency. Betaine status is influenced by dietary intake, endogenous synthesis from choline, BHMT-dependent metabolism, and renal handling. Lower circulating betaine concentrations and increased urinary betaine loss have been associated with obesity, insulin resistance, metabolic syndrome, and diabetes, suggesting that functional betaine insufficiency may accompany metabolic dysfunction [41]. From a translational perspective, baseline betaine status may be important for the response to supplementation. Patients with functional betaine insufficiency may benefit more from betaine supplementation than those with adequate betaine status. Therefore, future clinical trials should consider baseline betaine status and metabolic phenotype when evaluating the effects of betaine in MASLD.

## 4. Conclusions

Current preclinical evidence indicates that betaine modulates several mechanisms involved in the pathogenesis and progression of MASLD. In addition to regulating hepatic lipid metabolism, betaine improves insulin sensitivity, attenuates oxidative stress, endoplasmic reticulum stress, and mitochondrial dysfunction, inhibits inflammatory and fibrogenic signaling pathways, and regulates the gut–liver axis. However, evidence from human studies remains limited. Further experimental studies should define dose–response relationships and determine which molecular pathways are most responsive to betaine at physiologically and therapeutically relevant exposures. Available clinical findings suggest that the effects of betaine may depend on dose, treatment duration, and potentially the baseline metabolic and betaine status of the patient. Well-designed randomized controlled trials are required to establish effective dosing regimens, evaluate clinical efficacy and safety, and determine whether the mechanisms and benefits observed in preclinical models can be translated to patients with MASLD.

## Data Availability

No new data were created or analyzed in this study. Data sharing is not applicable to this article.

## Abbreviations

ACC	Acetyl-CoA carboxylase
Akt	Protein kinase B
AMPK	AMP-activated protein kinase
ApoE−/−	Apolipoprotein E-deficient
ATF6	Activating transcription factor 6
BGT1	Betaine/GABA transporter 1
BHMT	Betaine–homocysteine methyltransferase
CD36	Cluster of differentiation 36
ChREBP	Carbohydrate-responsive element-binding protein
CPT1	Carnitine palmitoyltransferase 1
CPR	Cytochrome P450 oxidoreductase
db/db	Leptin receptor-deficient diabetic mouse model
ECM	Extracellular matrix
eIF2α	Eukaryotic initiation factor 2 alpha
ER	Endoplasmic reticulum
EU	European Union
FASN	Fatty acid synthase
FDA	U.S. Food and Drug Administration
FGF10	Fibroblast growth factor 10
FGF21	Fibroblast growth factor 21
FOXO1	Forkhead box O1
FTO	Fat mass and obesity-associated protein
FXR	Farnesoid X receptor
GNMT	Glycine N-methyltransferase
GSK3β	Glycogen synthase kinase 3 beta
HCC	Hepatocellular carcinoma
HMGB1	High-mobility group box 1
HSCs	Hepatic stellate cells
IKKβ	Inhibitor of nuclear factor kappa B kinase beta
IL-1β	Interleukin 1 beta
IL-6	Interleukin 6
IL-18	Interleukin 18
iNOS	Inducible nitric oxide synthase
IRE1α	Inositol-requiring enzyme 1 alpha
IRS-1	Insulin receptor substrate 1
JAK/STAT	Janus kinase/signal transducer and activator of transcription
JNK	c-Jun N-terminal kinase
LPS	Lipopolysaccharide
LXRα	Liver X receptor alpha
m6A	N6-methyladenosine
MASH	Metabolic dysfunction-associated steatohepatitis
MASLD	Metabolic dysfunction-associated steatotic liver disease
MAT	Methionine adenosyltransferase
MCP-1	Monocyte chemoattractant protein 1
MMP-2	Matrix metalloproteinase 2
MMP-9	Matrix metalloproteinase 9
mTORC1	Mechanistic target of rapamycin complex 1
MTTP	Microsomal triglyceride transfer protein
MyD88	Myeloid differentiation primary response protein 88
NF-κB	Nuclear factor kappa B
NLRP3	NLR family pyrin domain-containing 3
NO	Nitric oxide
NRF-1	Nuclear respiratory factor 1
PDGF-BB	Platelet-derived growth factor BB
PEMT	Phosphatidylethanolamine N-methyltransferase
PERK	Protein kinase RNA-like endoplasmic reticulum kinase
PGC-1α	Peroxisome proliferator-activated receptor gamma coactivator 1 alpha
PI3K	Phosphoinositide 3-kinase
PKCε	Protein kinase C epsilon
PPARα	Peroxisome proliferator-activated receptor alpha
PPARγ	Peroxisome proliferator-activated receptor gamma
ROS	Reactive oxygen species
SAH	S-adenosylhomocysteine
SAM	S-adenosylmethionine
SCD1	Stearoyl-CoA desaturase 1
SCFAs	Short-chain fatty acids
sIgA	Secretory immunoglobulin A
SIRT1	Sirtuin 1
SLC6A12	Solute carrier family 6 member 12
SOCS3	Suppressor of cytokine signaling 3
SREBP-1c	Sterol regulatory element-binding protein 1c
T2DM	Type 2 diabetes mellitus
TFAM	Mitochondrial transcription factor A
TGF-β1	Transforming growth factor beta 1
TGR5	Takeda G protein-coupled receptor 5
TIMP-1	Tissue inhibitor of metalloproteinases 1
TLR4	Toll-like receptor 4
TNF-α	Tumor necrosis factor alpha
TonEBP/NFAT5	Tonicity-responsive enhancer-binding protein (nuclear factor of activated T cells 5)
TXNIP	Thioredoxin-interacting protein
UPR	Unfolded protein response
VLDL	Very-low-density lipoprotein
α-SMA	Alpha-smooth muscle actin
